# Urinary Casts

**Published:** 1903-10-31

**Authors:** 


### URINARY CASTS.

The exact significance of tube casts in the urine is a fruitful source of contention among both pathologists and clinicians, and it is no doubt, to some extent, on account of the want of more definite knowledge that the microscopical examination of urinary deposits is so frequently neglected in general practice. Dr. Z. Taylor Emery[1] summarises the present position of our knowledge of these products in a manner that should prove of much use for clinical purposes. He starts on the assumption that every tube cast has a hyaline basis, and he mentions that the hyaline substance is not a proteid, and that its composition is not known. He points out that the clinical significance to be attached to the presence of hyaline casts in the urine depends upon many qualifying factors and circumstances. The condition may be dependent upon circulatory disturbances, and these may be of a temporary or a permanent character. In other cases altered conditions of the blood are responsible, such for example as the circulation of irritating substances absorbed from the alimentary canal, or produced by defective metabolism, or by bacterial activity, as happens in the various fevers. All degrees may be found in these cases between the simple irritation, shown merely by the presence of hyaline casts, and definite inflammation with albuminuria and other signs of nephritis.

In those instances where hyaline casts are found but are small and are not accompanied by albumin, their presence need not be considered of much importance, especially if they are not present in large numbers. But if the casts are large, or of medium size and numerous, the condition should give rise to further examination and careful inquiry, especially as to the history of the patient, his habits with regard to occupation, eating and drinking, as well as the possibility of continued high blood-pressure from any cause.

Epithelium, blood or granular detritus may adhere to or become incorporated with hyaline casts either in the kidney, the ureters or the bladder, and the appearance of granular casts does not therefore always indicate an active destructive process within the kidney. It is necessary before pronouncing an opinion on such a case to obtain a complete clinical history of the patient and to make repeated chemical and microscopical examinations of the urine. The presence of albumin in addition to casts is of serious import if it is long continued and is not due to some temporary cause such as exhausting or violent exercise. Large hyaline casts, especially in the urine of middle-aged men of strenuous habits, if long continued, may be the earliest sign of interstitial nephritis. The method of examining for casts which Dr. Emery recommends is to filter five or six ounces of urine, then to puncture the filter paper and wash down any deposit there may be with some more urine into a centrifugal tube, and, after centrifugalising, to examine the deposit microscopically, without the aid of artificial light if possible.

[1] Brooklyn Med. Jour., Oct., 1903.

